# Genetic Landscape of Myeloproliferative Neoplasms with an Emphasis on Molecular Diagnostic Laboratory Testing

**DOI:** 10.3390/life11111158

**Published:** 2021-10-30

**Authors:** Arti Easwar, Alexa J. Siddon

**Affiliations:** 1 Department of Laboratory Medicine, Yale School of Medicine, New Haven, CT 06510, USA; arti.easwar@yale.edu; 2 Department of Pathology, Yale School of Medicine, New Haven, CT 06510, USA

**Keywords:** hematopathology, myeloproliferative neoplasms, chronic myeloid leukemia, *BCR-ABL1*, molecular diagnostics

## Abstract

Chronic myeloproliferative neoplasms (MPNs) are hematopoietic stem cell neoplasms with driver events including the *BCR-ABL1* translocation leading to a diagnosis of chronic myeloid leukemia (CML), or somatic mutations in *JAK2*, *CALR*, or MPL resulting in Philadelphia-chromosome-negative MPNs with constitutive activation of the JAK-STAT signaling pathway. In the Philadelphia-chromosome-negative MPNs, modern sequencing panels have identified a vast molecular landscape including additional mutations in genes involved in splicing, signal transduction, DNA methylation, and chromatin modification such as *ASXL1*, *SF3B1*, *SRSF2*, and *U2AF1*. These additional mutations often influence prognosis in MPNs and therefore are increasingly important for risk stratification. This review focuses on the molecular alterations within the WHO classification of MPNs and laboratory testing used for diagnosis.

## 1. Introduction

Myeloproliferative neoplasms (MPNs) are characterized by clonal proliferation of hematopoietic precursors in the bone marrow. The WHO Classification of Tumours of Haematopoietic and Lymphoid Tissues classifies these myeloproliferative neoplasms as chronic myeloid leukemia, *BCR-ABL1*-positive (CML); chronic neutrophilic leukemia (CNL); polycythemia vera (PV); essential thrombocythemia (ET); primary myelofibrosis (PMF); chronic eosinophilic leukemia (CEL); and MPN–unclassifiable (MPN-U) [1]. CML was the poster child for MPNs, first described by Dameshek in 1951, but it was not until 1960 that the defining t (9;22) was identified as the driver by Nowell and Hungerford [2,3,4]. Similarly, the diagnostic criteria for PV, ET, and PMF include the exclusion of *BCR-ABL1* as well. These three entities have overlapping morphologic features and thus are together referred to as *BCR-ABL1*-negative myeloproliferative neoplasms or Philadelphia chromosome-negative myeloproliferative neoplasms (Ph- MPNs) [1,5]. With the identification of *JAK2*, which encodes Janus kinase 2, in Ph- MPNs, our understanding of the molecular and genetic basis for these diseases has substantially improved [6,7]. Subsequently, the identification of *MPL* and *CALR* mutations has further advanced our understanding and diagnostic criteria [8,9,10,11]. In the past decade, with the emergence of new technologies such as microarray-based gene expression profiling (GEP) and next-generation sequencing (NGS), we have gained better insight into the molecular pathogenesis of chronic myeloid neoplasms [12]. This exponential knowledge and increase in available data have helped in the formation of specific algorithms for diagnosis and prognosis. This information has also aided in personalized treatment plans, follow-up methodologies, and molecularly targeted therapy [12]. In this review, we attempt to classify the different molecular alterations that aid in the diagnosis and the prognostication of MPNs. After summarizing the molecular characterization of each entity, we also outline the different methodologies used in laboratory testing for these molecular alterations to highlight the role of the laboratory to aid in the diagnosis, prognosis, and therapeutic approach.

## 2. Molecular Characterization of Myeloproliferative Neoplasms

### 2.1. Chronic Myeloid Leukemia, BCR-ABL1 Positive

CML is an MPN that is characterized by the clonal proliferation of myeloid elements (predominantly granulocytes) resulting from a reciprocal translocation of the *ABL* gene (located on chromosome 9) and the breakpoint cluster region *BCR* gene (located on chromosome 22) [1,13]. This leads to the formation of the t(9;22)(q34.1;q11.2) Philadelphia chromosome or an abnormal chromosome 22, where the *BCR-ABL1* oncogene constitutively encodes an oncoprotein with increased tyrosine kinase activity [3,13,14]. Depending on the breakpoint location, transcription of different *BCR-ABL1* mRNA transcripts is initiated by the *BCR-ABL* oncogene [13]. In greater than 95% of patients with CML, the most common transcript subtypes are e13a2 (also known as b2a2), e14a2 (also known as b3a2), or simultaneous expression of both [15]. Multiple other transcript subtypes such as e1a2, e2a2, e6a2, e19a2, e1a3, e13a3, and e14a3 have been reported, but these occur sporadically [16,17]. These different subtypes of *BCR-ABL1* transcripts encode fusion proteins of different sizes with tyrosine kinase activity and can result in different phenotypes. The most commonly reported major breakpoint region p210 *BCR-ABL1* proteins are encoded by e13a2 and e14a2 mRNA transcripts, but the resulting p210 proteins have slightly different sizes and induce slightly different phenotypes—patients with e14a2 transcripts have significantly higher platelet counts compared to patients with the e13a2 transcript [18,19,20]. p230 *BCR-ABL1* proteins are encoded by the e19a2 transcript, and these patients present with more prominent neutrophilic maturation and/or increased platelet counts. While p190 *BCR-ABL1* proteins (caused by the minor breakpoint and encoded by e1a2 transcript) most frequently result in Ph+ Acute Lymphoblastic Leukemia/Lymphoma, they can rarely be seen as the main protein in CML. These patients present with monocytosis without basophilia, which can be misclassified as Chronic Myelomonocytic Leukemia, and have a higher risk of progressing to the lymphocytic blast phase [21,22]. It should also be noted that very low levels of the p190 transcript can be found in typical p210 CML, caused by alternative splicing of the *BCR* gene [23].

#### 2.1.1. Tyrosine Kinase Inhibitors (TKIs) and Transcript Levels

Along with inducing different phenotypic features, different transcripts may also have varying sensitivities to tyrosine kinase inhibitors (TKIs) [24]. Some small studies have shown that e1a2 transcripts are associated with a less favorable response to TKIs with a faster rate of disease progression to blast phase with a less favorable response and a faster disease progression [21,25,26]. In order to elucidate these findings, Gong et al. analyzed the risk and frequency of blast transformation, response to treatment with TKIs, and clinical outcomes in 2322 treated CML patients and corresponding transcripts, with the intention to elucidate the differences between e1a2 transcripts (p190) and the more common p210 breakpoint [15]. They concluded that patients with this mRNA transcript had a higher frequency of additional chromosomal abnormalities (ACA), significantly worse survival, a shorter period to blast transformation (which was lymphoid or mixed type), and a lower likelihood of achieving major molecular remission (MMR) after TKI treatment when compared to patients with e13a2 and e14a2 (p210) transcripts. This study also shows that the presence of e1a2 can be considered as high risk and patients would need additional therapy and close monitoring. Since both e13a2 and e14a2 are only different by the presence of 25 additional amino acids in the latter, there have been studies to further investigate the impact of each on clinical course, response to TKIs, and overall survival. In a study of 105 patients by Hanfstein et al., clinical presentation and response to treatment with and/or without TKIs were evaluated [20]. They observed that patients with e14a2 presented with lower leukocyte counts and higher platelet counts when compared to patients with e13a2. In terms of molecular response, e14a2 patients showed a faster response to TKIs when compared to patients with e13a2. Similar conclusions were found by additional studies [27,28]. In a more recent study at MD Anderson Cancer Center (MDACC), 481 patients were enrolled to assess the impact of transcript type on different TKI therapies, which included imatinib (at two dosages) and second-generation TKIs, dasatinib, and nilotinib [18]. The authors found that patients with e13a2 transcripts treated with lower dose imatinib had the longest time to molecular remission when compared to treatment with higher dose imatinib or second-generation TKIs. Patients with e14a2 transcripts who were treated with second-generation TKIs achieved faster and longer-term cytogenetic and molecular responses when compared to patients with e13a2 transcripts. On the other hand, results were similar in patients with co-expression of e14a2 and e13a2 transcripts who were either treated with low-dose imatinib, high-dose imatinib, or second-generation TKIs. They hypothesized that since e13a2 has higher tyrosine kinase activity than e14a2 transcripts, higher-dose imatinib and second-generation TKIs are better equipped to reduce tyrosine kinase activity than low-/standard-dose imatinib [18].

#### 2.1.2. ABL1 Kinase Domain Mutations

Since the advent of TKIs, the management and treatment of patients with CML have significantly improved with dramatically reduced rates of stem cell transplantation [29]. TKI therapy is monitored by assessing *BCR-ABL1* transcript level to achieve Major Molecular Remission [30]. Currently, the European LeukemiaNet (ELN) recommends the use of a first-generation TKI (imatinib) as the first-line treatment [30,31]. However, emerging TKI resistance has complicated long-term management of these patients. The most common cause of first-generation (Imatinib) TKI resistance is the presence of *ABL1* kinase domain (KD) mutations, which include M244V, G205E, Q252H, Y253F/H, E255K/V, D276G, F311L, T315, F317L, M351T, E355G, F359V, L384M, L387F, H396R/P, E459K, and F486S, but many other missense mutations have been identified [29,32,33]. These mutations occur in leukemic stem cells and are generally point mutations in the *BCR-ABL1* kinase domain. T315I is considered a gatekeeper mutation as it is resistant to all TKIs other than ponatinib [33]. Previously done by Sanger sequencing, currently, NGS is the preferred methodology for resistance mutation detection, with better sensitivity [32].

If complete or deep molecular response (CMR), which is defined as *BCR-ABL1* transcripts ranging from ≤ 0.01% (MR^4^) to ≤ 0.001% (MR^5^), is not achieved or if the response is suboptimal, it is imperative to assess for *BCR-ABL1* KD mutations [34,35]. Similarly, after initial treatment, it is recommended that these patients undergo NGS for mutational testing as well. Resistance mutations are not as common in the chronic phase as in the advanced phases and if any mutations are identified, treatment with next-generation TKIs is initiated [34,35].

KD mutations account for about 30–90% of cases of TKI resistance [36]. In order to understand any other causes of TKI acquired resistance, Schnittger et al. investigated pan-myeloid markers in established cases of TKI-resistant CML using NGS. They observed that the most common cause of primary TKI resistance was mutations in *ASXL1*, which was already present in these patients at the time of CML diagnosis. They also noticed that the *ASXL1* mutational load in these patients correlated with *BCR-ABL1* transcripts. Other genes identified were *RUNX1*, *IDH1,* and *DNMT3A* [36]. However, the impact of such genes is not entirely elucidated [37].

#### 2.1.3. Disease Diagnosis and Laboratory Monitoring

As outlined by ELN and the National Comprehensive Cancer Network (NCCN) guidelines, the current gold standard to monitor treatment is the use of quantitative reverse-transcriptase PCR (RQ-PCR) to quantify the *BCR-ABL1* transcripts periodically, starting with 3-month intervals [30,31,34,38,39,40]. While monitoring is most often done in peripheral blood, bone marrow cytogenetics are suggested by the NCCN guidelines at diagnosis, with failure to reach response milestones, increase in *BCR-ABL1* transcript to >1%, or suggestion of loss of hematologic response [38]. Major molecular response (MMR) is achieved when transcript levels are ≤0.1%, while a complete or deep molecular response is achieved when transcript levels are ≤0.01%. The current recommendations are to test the patient at 3-month intervals while on treatment with TKIs until MMR and subsequently CMR is achieved [40,41]. This applies to 90–95% of CML cases with the Ph chromosome, which is also detected with chromosomal analysis as well as using fusion probes in fluorescence in situ hybridization (FISH) at the time of diagnosis [40]. However, 5–10% cases can have variant t(9;22) which can either involve additional chromosomes or have cryptic gene rearrangements, leading to the formation of *BCR-ABL1* gene fusion [18]. Additionally, some CML patients can also have ACA such as an additional Ph chromosome, trisomy 8, isochromosome 17q, trisomy 9, abnormalities of 3q26.2, or a complex karyotype [1,40]. Such abnormalities can arise both during the course of disease progression or during therapy and warrants cytogenetic studies for accurate analysis. Such ACAs are in fact included in the WHO 2017 criteria for defining the accelerated phase of CML and have been shown to be independent prognostic factors to predict disease progression and overall poor survival [18,42]. Just as RQ-PCR is used to monitor transcript levels during and after therapy, cytogenetic studies can be used to monitor the level of Ph+ cells. This method also helps detect any additional clonal abnormalities that may arise during the course of therapy [41]. Some studies have shown that some new clonal abnormalities may not be of clinical significance, but CML patients harboring additional chromosomal abnormalities such as 7q deletions or monosomy 7q can indeed develop myelodysplastic syndrome (MDS) or acute myeloid leukemia (AML) [43,44]. Thus, MRD during and after therapy should include transcript detection by PCR (which can be either on bone marrow or peripheral blood samples), karyotype/cytogenetic studies, and NGS in cases of suspected KD mutations [40,41]. Kinase domain mutations are most commonly T315 mutations and can be detected with a sensitivity of 20% by Sanger sequencing and approximately 3% by NGS. Kinase domain mutational analysis should be assessed with loss of hematologic response, >1-log increase in *BCR-ABL1* transcript levels, and loss of MMR, or if there is disease progression to accelerated or blast phase [31].

### 2.2. Philadelphia Chromosome-Negative Myeloproliferative Neoplasms (Ph- MPN)

Ph- MPNs consist of three different entities, Essential Thrombocythemia (ET), Polycythemia Vera (PV), and Primary Myelofibrosis (PMF), which are all characterized by overproduction of differentiated cells of various lineages, which can typically be identified in the peripheral blood [1,45]. All three entities have an increased risk of thromboembolic complications—both venous and arterial, hemorrhage, and progression to acute myeloid leukemia [1]. Even though these neoplasms each have distinct characteristics, clinically, there can be much overlap, making the diagnosis challenging; both polycythemia vera and essential thrombocythemia can progress into secondary myelofibrosis [1,45].

The genetic hallmark of Ph- MPN is the constitutive activation of the JAK-STAT pathway [46,47]. Hematopoietic stem cells (HSCs) can acquire somatic driver mutations, which leads to the formation of mutant proteins that affects the JAK-STAT pathway and results in uncontrolled cell proliferation in all three lineages [12,46]. JAK2 is associated with cytokine receptors such as erythropoietin receptor (EpoR), thrombopoietin receptor (TpoR), and granulocyte-colony-stimulating factor receptor (G-CSFR) [12]. TpoR is encoded by the myeloproliferative leukemia virus oncogene (*MPL*) and G-CSFR by Colony-Stimulating Factor 3 Receptor (*CSF3R*). Driver mutations that affect this pathway are predominantly *JAK2* V617F and *JAK2* exon 12 mutations, *MPL* mutations, *CALR* exon 9 mutations, and mutations in *CSF3R* [8,9,45,48,49,50,51,52,53]. The most commonly implicated driver mutations in MPNs are listed in Table 1. Other genes that function as negative regulators of the JAK-STAT pathway have also been identified; however, in MPNs, these have been shown to have lower frequency of inactivation [45]. Such negative regulators include SH2B3 (lymphocyte-specific adaptor protein or LNK) and *CBL* (casitas B-lineage lymphoma proto-oncogene) [54,55,56]. Megakaryocytic differentiation and platelet production are further regulated by TPO and TpoR (MPL)/JAK2 axis. The main mutations affecting this pathway are in *MPL*, *THPO,* and *JAK2* [57].

#### 2.2.1. Polycythemia Vera

PV presents with erythrocytosis in the peripheral blood and is suggested clinically by hemoglobin levels greater than 16 g/dL in women and greater than 16.5 g/dL in men, along with hyperplasia of all the marrow lineages resulting in panmyelosis, but may be most morphologically apparent by increased megakaryocytes and erythroid precursors [1,45]. The presence of the *JAK2* V617F mutation is diagnostic and is present in greater than 95% of cases; however, approximately 3–4% of cases are associated with various *JAK2* exon 12 mutations, which are considered functionally similar, resulting in an increased RBC production [5,46,58]. Rare cases that are *JAK2*-negative harbor either driver mutations that activate the JAK-STAT pathway or non-driver mutations involved in DNA methylation [46,58]. A small percentage of *JAK2*-negative cases have shown mutations in genes involved in histone modification as well [46].

#### 2.2.2. Essential Thrombocythemia

ET presents with platelet counts greater than 450 × 10^9^ /L and morphologically atypical megakaryocytes, with no or minimal increase in marrow cellularity [1,59]. Leukocytosis and erythrocytosis are rare but have been reported. Patients generally present at 50–60 years of age (with another incidence peak at 30) with elevated platelet counts, but since there are no genetic markers specific for ET, other causes of thrombocytopenia such as inflammation/infection, PV/PMF, or other neoplasms have to be excluded as well. ET in children is very rare but has been reported and must be distinguished from hereditary erythrocytosis, which involves germline mutations in *JAK2* or GSN (gelsolin gene) [1].

Genetically, 50–60% of ET patients harbor the *JAK2* V617F (or functionally similar) mutations, while 15–30% patients present with *CALR* and 1–4% have the *MPL* mutations [1,60]. However, up to 12% of cases are triple-negative for these driver mutations, but with the advent of whole-exome sequencing, additional gain of function mutations in the *MPL* gene have been identified [1,60].

#### 2.2.3. Primary Myelofibrosis

PMF is the proliferation of bizarre megakaryocytes and myeloid hyperplasia within the bone marrow, with evolving bone marrow fibrosis, until the overt stage where patients present with leukoerythroblastosis and extramedullary hematopoiesis, predominantly in the liver and spleen, resulting in hepatosplenomegaly [1]. Molecularly, approximately 50–60% of cases harbor *JAK2* V617F mutations, 20–24% have *CALR* mutations, and 8–10% have *MPL* mutations. However, about 12% of cases have none of these and are regarded as “triple-negative”. With the increased use of NGS, multiple other cooperating mutations in genes associated with myeloid malignancies have been identified, including *ASXL1*, *EZH2*, *TET2*, *IDH1*, *IDH2*, *SRSF2*, and *SF3B1* [1,61]. More recently, *SRSF2*, *ASXL1,* and *U2AF1*-Q157 mutations have been shown to predict poor survival independent of other risk factors [61]. Multiple risk assessment models have been in use to assess prognosis in PMF patients. The most commonly used model is the IPSS-R (international prognostic scoring system), which is designed to be used at the initial time of diagnosis and uses five independent factors to predict survival. DIPSS (dynamic IPSS) assigns additional points to lower hemoglobin levels and classifies risk as low, intermediate, and high. More recently, however, mutations have also been incorporated into developing three prognostic models—mutation enhanced IPSS for patients 70 years or younger (MIPSS70), MIPSSv2 (MIPSS version 2.0), and genetically inspired prognostic scoring system (GIPSS). MIPSS70, which is used in patients who are transplant-eligible, includes three genetic and six clinical risk factors. The genetic variables include the absence of *CALR* type-1-like mutations, the presence of high-risk mutations such as *ASXL1*, *SRSF2*, *EZH2*, *IDH1,* or *IDH2*, and the presence of two or more high-molecular-risk mutations. MIPSSv2 uses mutations, karyotypes, and clinical variables to assess prognosis. Additionally, this model uses *U2AF1* Q157 as a high-molecular-risk mutation. Karyotype, very high-risk mutations, and the absence of *CALR* type 1-like mutation and other clinical variables have been included in this prognostic model [61]. GIPSS, on the other hand, exclusively uses karyotype and mutations [62].

### 2.3. Driver Mutations in Ph- MPNs

#### 2.3.1. JAK2 Mutations

*JAK2*, located on the short arm of chromosome 9, encodes the non-receptor tyrosine kinase Janus kinase 2 (JAK2) which is associated with cytokine receptors [48,63]. Upon ligand (cytokine) binding, JAK2 is phosphorylated and activates downstream proteins that are involved in the regulation of gene expression and cell proliferation [63]. *JAK2* is an important mediator of erythropoiesis and is the most frequent gene implicated in *BCR-ABL1*-negative myeloproliferative neoplasms [48].

The most common *JAK2* mutation encountered in PV is the V617F somatic mutation in exon 14, which results in the substitution of valine to phenylalanine at position 617 in the pseudokinase domain [46,47,48,63]. This mutation results in a JAK2 conformational change and causes cytokine-independent activation of the JAK-STAT pathway, which constitutively activates downstream targets, resulting in uncontrolled clonal proliferation, and an increase in predominantly normal red blood cells [48,63,64]. The *JAK2* V617F mutation arises in a multipotent hematopoietic stem cell and thus is present in all myeloid and lymphoid (B and NK) cells, with a later rare presentation in T cells [46].

In *JAK2* V617F-negative cases of PV, *JAK2* exon 12 mutations have been identified [5,46,48]. These mutations consist of duplications or substitutions but are commonly in-frame insertions or deletions [46,48]. The most frequently detected mutations are N542-E543del, E543-D544del, and rarely K539L [46,48]. These mutations affect the amino acids between the SH2 and pseudokinase domains, which leads to constitutive kinase activation [48]. Clinically, some studies have shown that patients with *JAK2* exon 12 mutations are younger and present with erythrocytosis, but have similar symptoms and rates of survival when compared to patients with *JAK2* V617F mutations [48].

*JAK2* mutations are not specific for PV and can be identified in up to 60% cases of ET and PMF [5,46,63]. Mitotic recombination can result in a copy-neutral loss of heterozygosity (LOH) along a variable size region on 9p, which can be observed in both ET and PV [5]. However, the variant allelic frequency (VAF) is higher in PV (50%) when compared to ET (25%) and 100% in post-ET or post-PV myelofibrosis [5]. This was also shown in a study by Tiedt et al., where low *JAK2* V617F expression levels correlated with an ET-like phenotype, while high expression levels were shown to induce a PV-like phenotype in mouse models [12,65]. *JAK2* V617F can also be detected in the general population at very low levels or in refractory anemia with ring sideroblasts and thrombocytosis (RARS-T) or in clonal hematopoiesis of indeterminate potential (CHIP) [66,67,68].

Thrombotic complications, which include venous thromboembolism or arterial thrombosis, occur more commonly in about 12–39% of PV patients but can also be seen in a small percentage of ET patients [69]. In fact, up to 30% of PV cases are diagnosed after myocardial infarctions [70]. Other serious events include abdominal vein thrombosis, Budd–Chiari syndrome, and obstruction of portal, mesenteric, and splenic vascular systems. Factors implicated in this increased risk of thrombosis include age over 60 years and any known history of thrombosis [1]. Other known complications are progression to post-PV Myelofibrosis (MF) and acute myeloid leukemia (AML). Leukocytosis greater than 15 × 10^9^/L and longer duration of disease are both implicated in predicting the risk of post-PV MF, while leukocytosis (>15 × 10^9^/L) and advanced age are poor prognostic factors implicated in progression to AML [1,71]. Multiple studies have tried to determine if *JAK2* V617F allelic burden influences the risk of thrombotic events, not just in PV but in other *BCR-ABL1*-negative myeloproliferative neoplasms (Ph- MPNs) as well. A prospective study by Passamonti et al. looked at *JAK2*-mutated cases of PV and compared allelic burden with multiple factors such as hemoglobin concentration, WBC count, bone marrow cellularity, size of spleen, and platelet counts. They analyzed the allelic burden in 320 cases of PV with the *JAK2* V617F mutation and found that higher allelic burden was directly related to hemoglobin concentration, marrow cellularity, splenomegaly, and WBC counts but inversely related to platelet counts. The study concluded that the *JAK2* V617F mutant allelic burden greater than 50% was significantly related to the risk of developing post-PV MF but not to the risk of developing AML or thrombosis [71]. In contrast, in a retrospective study by Borowczyk et al. in 2014, *JAK2* V617F allelic burden in 126 patients with Ph- MPN and the mutation was analyzed with the intention of assessing its role in the risk stratification of future vascular complications. They quantified the allelic burden according to percentages: levels >51% were considered homozygous for the mutation, and the highest levels were found in patients with PV. They concluded that as allelic burden increased the risk of venous thromboembolism increased as well. At allelic burden greater than 25%, they observed a 7.4-fold increase in the risk of thrombosis as compared to patients with <25% *JAK2* V617F allelic burden [69].

#### 2.3.2. CALR Mutations

*CALR* is the second most frequently mutated gene encountered in Ph- MPNs, although these mutations have not been identified in PV patients [63]. *CALR* mutations were recently discovered in 2013 by two separate studies that utilized whole-exome sequencing [8,9]. Both studies found recurrent mutations in *CALR* in 70–80% ET and PMF patients who were negative for the two known driver mutations, *JAK2* and *MPL*. In an early study by Klampfl et al., amongst 1107 patients with MPN, *CALR* mutations were identified in 25% ET and 35% PMF patients, but not in PV patients or other myeloid neoplasms [8]. *CALR*, *JAK2*, and *MPL* mutations are considered to be mutually exclusive; however, recently, few cases with concurrent *JAK2* V617F, *MPL*, and/or *CALR* mutations have been reported [72,73,74,75,76].

*CALR*, which encodes a highly conserved calcium-binding chaperone protein calreticulin, is located on chromosome 19 [49]. Calreticulin is located on the endoplasmic reticulum (ER) through the C-terminal ER-retention sequence KDEL, and it was hypothesized that mutations implicated in ET and PMF affect the KDEL motif and subsequently affect the protein’s localization on the ER; however, recent studies with exogenous mutated *CALR* have shown that it retains localization to the endoplasmic reticulum showing that there are other KDEL-independent mechanisms as well [49,63,77]. *CALR* has multiple functions—maintaining calcium homeostasis and assisting in protein folding, cell adhesion, immune response, and phagocytosis [63,77]. More than fifty different mutations, all in exon 9, are implicated in ET (20–25%) and PMF (25–30%) [77]. Two specific mutations account for about 80% of described subtypes: type 1, more common in Ph- MPN (53%), a 52bp deletion (L367fs*46), and type 2, in about 32% cases, a 5bp insertion (K385fs*47). Both of these result in the same C-terminal amino acid sequence [63,77]. Further, these mutations result in mutant proteins with loss of negatively charged amino acids, which affects the KDEL motif [77,78]. The remainder of *CALR* mutations in ET and PMF are classified as type 1-like or type 2-like, depending on their corresponding similarities in structure, extent of amino acid deletions, and effect on the KDEL motif at the C-terminus [45,49,77].

Clinically, different mutations have distinguishing presentations as well. In one study by Rumi et al., driver mutations in 1135 patients with either PV or ET were analyzed. For *CALR*-driven ET cases, mutated calreticulin had a lower calcium-binding affinity and showed a loss of the ER retention KDEL motif [79]. The allelic burden was calculated and when compared to *JAK2*-mutated ET, *CALR* allelic burden was higher. Clinically, *CALR*-mutated ET patients have much higher platelet counts (>1000 × 10^9^/L) and presented at a younger age; however, their risk of thrombosis was lower than patients with *JAK2* mutated ET [79]. Similarly, patients with *CALR*-mutated PMF have been shown to have a more indolent clinical course [76,78,80]. Further, PMF patients with *CALR* mutations have a better overall survival when compared to *JAK2-* or *MPL*-mutated cases [8]. Tefferi et al. studied 254 PMF patients and concluded that patients with *CALR* mutations were younger, with a higher platelet count, and presented with lower DIPSS-plus score; additionally, they were also less likely to require blood transfusions for anemia or present with leukocytosis [76].

#### 2.3.3. MPL Mutations

*MPL* mutations were the second driver mutations to be identified in *JAK2*-negative MPNs [10,61,81]. Myeloproliferative leukemia virus (*MPL*) is located on chromosome 1p34 and consists of 12 exons that encode the 70kDa thrombopoietin receptor protein (TpoR) [57]. This receptor is a homodimer and is composed of three domains: the extracellular domain binds thrombopoietin (TPO), transmembrane domain, and the intracellular domain, which binds to JAK2, leading to phosphorylation of MPL and activation of multiple downstream signaling pathways within the JAK-STAT pathway and affecting megakaryopoiesis [57,82]. Mutations in *MPL* have been associated with both thrombocytosis and thrombocytopenia [57]. The first *MPL*-activating mutation in exon 10, *MPL* W515L, was discovered in 2006 in a *JAK2*-negative ET/PMF [10,61]. *MPL* W515L mutation results from G to T substitution at position 1544, leading to a leucine residue at codon 515 [57,63,77,82]. The presence of tryptophan is part of an amphipathic environment in the junctional area between the transmembrane and intracellular domains of TpoR [57]. This replacement leads to MPL dimerization, inducing constitutive activity independent of TPO [83]. When *MPL* W515L retroviral mouse models were bone-marrow-transplanted, Pikman et al. observed that these mouse models presented with thrombocytosis, which was followed by myelofibrosis, a similar evolution of disease observed in ET [10]. Other common mutations identified in ET and PMF appear to cluster in exon 10 and affect the same W515 residue (W515K–second most common, W515A, W515G, and W515R) [57,63,83]. Such activating mutations are found in 1–4% ET and 5–10% PMF patients [63,77,82]. However, *MPL* W515 mutations are not only found in MPNs, they have also been described in RARS-T [84,85].

*MPL* variants outside of exon 10 have also been identified in sporadic MPN cases. Such acquired mutations result in amino acid substitutions in either the extracellular (S204P/F, E230G) or intracellular (Y591D/N, T119I) domains of the MPL/TpoR but have shown a lower gain-of-function activity when compared to *MPL* W515L mutants [46,57,86,87]. These can also be associated with other acquired mutations in *JAK2*. Another rare somatic mutation that has been reported in less than 1% of ET and PMF cases is *MPL* S505N, which is part of the TpoR transmembrane domain, and the mutation leads to constitutive dimerization of MPL/TpoR, leading to activation of the downstream signaling pathways [46,57,84]. This gain of function mutation was originally reported as a germline mutation implicated in hereditary thrombocytosis [46,57,88]. Germline gain-of-function and loss-of-function mutations in both *MPL* and *THPO* genes have been identified in hereditary thrombocytosis as well as inherited thrombocytopenia [46,57]. Thrombocytosis with *MPL* or *THPO* germline mutations are a result of gain-of-function mutations, which lead to defective MPL/TpoR, either because of loss of signaling or impaired stability, which constitutively activates the MPL signaling pathways. Similarly, thrombocytopenia results from loss-of-function germline mutations and leads to no production of TPO or impaired binding of MPL to TPO, impairing the MPL signaling pathways and decreasing platelet production [57,89,90,91,92]. It is imperative to distinguish such familial/hereditary cases from sporadic mutations in the *MPL* genes in cases of ET.

## 3. Molecular Diagnostics for Ph- MPNs

The current WHO classification of Ph- MPNs does not require the identification of a driver mutation in order to make a diagnosis; however, they are typically a major criterion because morphology and clinical presentation alone can overlap with reactive myelopoiesis [93]. The presence of driver mutations with or without additional non-driver somatic mutations, as listed in Table 2, along with relevant morphologic features, can support the diagnosis of Ph- MPNs [63,93].

### 3.1. Detection of Driver Mutations

Currently, there are no Food and Drug Administration (FDA)-approved detection methods for driver mutations [94]. For *JAK2* V617F mutations, the most commonly used method is real-time allele-specific PCR (rt-PCR) [63,94]. The sensitivity of rt-PCR ranges from 0.1 to 1%, but the specificity is 100% [63,93,94,95,96,97]. Quantitative PCR (qPCR) is preferred over qualitative methods because of better reproducibility and the ability to monitor burden [63,94]. Droplet digital PCR (ddPCR) is now used to accurately quantify mutants of the target genes with very high sensitivity [98,99,100]. It has also been shown that about 10% of the general population can have *JAK2* V617F mutation when detected by rt-PCR [101]. However, not all these patients develop Ph- MPNs, and thus it may be difficult to distinguish patients with Ph- MPN and low allelic burden and healthy patients with low-level *JAK2* V617F mutations without any known clinical significance [102].

*JAK2* exon 12 mutations are rare, and many of the mutations detected are of uncertain clinical significance [103,104]. Hence, Sanger sequencing for these mutations has most frequently been used; however, nested High-Resolution Melting (HRM) has been proposed as an initial screening step before sequencing in order to characterize the mutations more precisely [94,103,104,105].

*CALR* exon 9 mutations are more commonly detected by fragment analysis, which has relatively high sensitivity and also evaluates mutant allelic burden [8,79,106,107]. Another commonly used screening technique is HRM analysis followed by either qPCR or ddPCR [63]. However, Sanger sequencing is considered the most precise technique to detect and determine the genotype of all the different mutations that can be detected in positive samples. Another important issue is to realize that multiple single nucleotide polymorphisms and in-frame germline mutations have also been identified, which can be interpreted as driver mutations if they are not correctly characterized, highlighting the importance of variant interpretation by experienced laboratorians [108]. Another alternative method to detect *CALR* exon 9 mutations is the use of a standardized PCR-HRM analysis method or fragment analysis combined with Taqman Real-Time quantitative PCR (RT-qPCR) [109]. Even though next-generation sequencing (NGS) is considered to be the most accurate method with a sensitivity of 2–5%, given the heterogeneity and the high allelic burden of *CALR* mutations, such a sensitive method may not be necessary for driver mutation detection in routine cases [63,94]. Nevertheless, Sanger sequencing remains the most frequent and primary method of testing to detect mutations in exon 9 and their corresponding specific sequence changes (insertions or deletions) [63].

The most frequent *MPL* mutations are detected in exon 10, and the methods of detection for these mutations depend on targeted identification of the individual mutations or sequencing of exon 10 entirely [63]. Identification of specific mutations would require several assays to detect them individually using allele-specific qPCR (AS-qPCR), and the analytical sensitivity reported by this method is the highest at 0.1–0.5% in multiple studies [84,110,111,112,113]. A multiplexed AS-qPCR assay, which can detect the four most commonly detected *MPL* mutations, namely W515L, W515K, W515A, andS505N, has been developed [114]. The specificity for multiplexed AS-qPCR is 100% with the analytical sensitivity approximating 2.5%. On the other hand, the advantage of sequencing the entire exon, however, is that all known mutations as well as novel mutations can be detected; however, the analytical sensitivity by this method has been reported to be 2–5% [110,114,115].

With the advent of genome-wide NGS, our understanding of the different genetic alterations involved in human cancers has exponentially increased. NGS has several advantages over traditional sequencing methods of detection as well. First, NGS is a high-throughput method of analysis that can detect multiple different mutations in the same run. Secondly, this mode of detection does not require high-input DNA/RNA samples and also has much higher sensitivity (<1%). NGS can also detect many other genomic deviations such as single-nucleotide variants (SNVs), insertions, deletions, and copy number variations (CNV) with high accuracy. The gene fragments detected through different available NGS platforms are then compared to the human reference genome through bioinformatics analyses [63,116]. Thus, NGS can detect known as well as novel mutations in the gene panels that are tested.

### 3.2. Detection of Somatic/Acquired Non-driver Mutations or “Cooperating Mutations”

The vast majority of Ph- MPNs can be attributed to the three known driver mutations. However, the heterogeneity of PH- MPNs cannot be explained by mutations in just these three genes. Ph- MPNs have often been shown to contain at least one additional non-driver mutation when samples were analyzed with NGS. Additional mutations have been detected in about 50% of ET and PV patients and 80% of PMF patients [117,118] These additional mutations, also termed cooperating mutations, increased in number as disease progressed. Mutations identified were epigenetic regulators, which include DNA methylation regulators (*TET2*, *DNMT3A*, and *IDH1/2*) and histone/chromatin modifiers (*ASXL1* and *EZH2*), splicing factors (*SRSF2*, *SF3B1*, *U2AF1*, *ZRSR2*), signal transduction factors (*SH2B3/LNK* and *CBL*), and transcription factors (*TP53* and *RUNX1*) [46,93,117,118]. These mutations are not specific to MPNs and have been implicated in other myeloid malignancies, including Myelodysplastic syndromes and AML as well. Some of these mutations have also been reported in a small proportion (5%) of healthy adults, termed Clonal Hematopoiesis of Indeterminate Potential (CHIP) [119]. These genes and their prognostic implications are summarized in Table 2.

Delic et al. studied 100 cases of MPNs and concluded that splicing genes (*SRSF2*, *SF3B1,* and *U2AF1*) were most frequently mutated in PMF [120] and were either rare or not at all in ET and PV, respectively. Additionally, epigenetic regulator genes (*ASXL1*, *EZH2*, *TET2*, *DNMT3A,* and *IDH1/2*) were more frequently mutated in PMF patients than in ET or PV patients. Out of these, *ASXL1* and *TET2* were associated with poor survival, which was in accordance with other studies [121,122].

*TET2* and *DNMT3A* play a key role in DNA methylation regulation by methylating (DNMT3A) and the demethylating (TET2) at cytosine residues in the cytosine-guanine (CpG) dinucleotide sequences [46,93,123]. TET2 was one of the first mutations to be identified in *JAK2* positive MPNs and is identified in about 10–15% MPNs [123]. Some studies have shown that patients with *TET2* mutation have progressed to AML; however, other studies have not shown a clear increase in the risk of thrombosis or overall poor survival in MPN patients [121,124,125]. *DNMT3A* mutations only occur in about 5–10% of MPNs and mostly precede *JAK2* or *MPL* mutations [126]. R882H is the most frequent mutation encountered, and this mutation has been shown to induce myelofibrosis and AML transformation when present with *JAK2* V617F [126,127]. Both mutations result in increased self-renewal of *JAK2* mutated hematopoietic stem cells, thus playing a key role in disease initiation; however, if they occur as secondary mutations, they aid in disease progression [46]. Similar to *TET2*, patients with *DNMT3A* mutation first are more likely to develop ET-like phenotype, while patients with *JAK2* first are more likely to develop PV-like phenotype [122,128]. *IDH1/2* genes are also included in this group. Mutations in these genes commonly occur at R132 (*IDH1*) or R140/R172 (*IDH2*) and result in the inappropriate conversion of a-ketoglutarate to 2-hydroglutarate, leading to dysregulation of downstream targets affecting leukemogenesis [129,130,131]. Such mutations have been reported in about 5% of MPNs but in 20–25% MPNs in blast crisis/phase. This shows that the presence of *IDH1/2* mutations can predict progression to myelofibrosis and AML transformation [93,129,132]. Within the histone modifiers, two frequently mutated genes have been identified in PMF: *ASXL1* and *EZH2*. *ASXL1* or additional sex combs-like 1, interacts with Polycomb group complexes (PgC), which are involved in histone modifications to continue gene expression [46,133,134]. Many studies have shown the importance of *ASXL1* in normal hematopoiesis, but along with *TET2*, this gene is the second most common to be implicated in MPNs [46]. Most mutations identified in *ASXL1* are frameshift or nonsense mutation (exon 12) in the N-terminal, which results in a C-terminal truncated protein that affects the subsequent binding of BAP1, which binds to chromatin and brings about additional epigenetic modifications [46,134]. Such mutations are found in 30–40% PMF patients and about 5–10% of PV/ET patients [93]. *EZH2* encodes for a histone methyltransferase, which also interacts with the PgC and is normally involved in the suppression of H3K27 trimethylation [46]. Loss of function mutations and cytogenetic abnormalities in *EZH2* are commonly identified in 5–10% PMF patients, but such mutations have also been identified in other myeloid malignancies [135,136,137]. Both *ASXL1* and *EZH2* mutations can occur as early events in hematopoietic stem cells and can exist simultaneously in the same clone. Patients with such double mutations presented with decreased hemoglobin levels and increased WBC counts. Both mutations are also associated with an increased risk of transformation to MF and AML, thus indicating a poor clinical outcome, especially when present along with *JAK2* V617F [137,138,139,140].

The second group of mutations involves spliceosome/splicing genes, which are detected in 3–5% MPNs, with a higher frequency in PMF [117,141]. Genes implicated in MPNs within this group are *SF3B1*, *SRSF2,* and *U2AF1*. Most documented mutations in this group are heterozygous missense mutations, which lead to altered function and can affect RNA splicing [5,142]. SF3B1 mutations have been reported in about 10% of MPNs, more commonly in PMF or Post-ET/PV MF [142,143]. This gene encodes for the largest subunits of splicing factor 3b, the core component of spliceosomes [144]. Somatic mutations result in altered splicing of pre-mRNA, which leads to an accumulation of functionally defective proteins, phenotypically leading to cytopenias [5,145]. SF3B1 mutations have been largely associated with myelodysplastic syndromes and are associated with the presence of ring sideroblasts but have also been associated with disease progression to post-ET MF [143]. *SRSF2* mutations have been reported in up to 20% of PMF [93]. This gene encodes for serine and arginine rich splicing factor 2, which contains an RNA-recognition motif that facilitates spliceosome assembly [146]. Mutations in this gene also result in the downregulation of *EZH2,* which subsequently leads to myelofibrosis and overall poor survival [5,141,146,147]. U2AF1 is another gene that is involved in splice site recognition [148,149]. Mutations in these genes have been associated with AML transformation in MPN cases [5,46].

Tefferi et al. compared the clinical and molecular features of 254 patients with PMF. These patients were screened for *ASXL1*, *EZH2*, *IDH1/2*, *SRSF2,* and *U2AF1*. The mutational frequencies of these cooperating genes were highest in *ASXL1* (31%), *U2AF1* (16%), *SRSF2* (12%), *SF3B1* (7%), *EZH2* (6%), and *IDH* (4%) [76]. Based on their data, they concluded that *CALR*-mutated patients have favorable prognostic features such as decreased anemia and leukocytosis, while *CALR*-/*ASXL1*+ have decreased survival. They also identified that the frequency of *U2AF1* mutations was lower in *CALR*-mutated PMF cases but higher in *CALR*-mutated ET cases, which may account for the opposing trends in anemia [8,9,53,150,151]. These results support the classification of *ASXL1*, *SRSF2*, *EZH2*, *IDH1/2*, and *U2AF1* as high-risk mutations and their inclusion in the MIPSSv2 prognostic model for risk stratification in PMF [61,76,152].

Another important gene that has been implicated in *JAK2* negative PV cases is *LNK,* which encodes for the lymphocyte adapter protein [153]. *LNK* (also known as *SH2B3*) plays an important role as a negative regulator of the JAK-STAT and the TPO/MPL pathways and thus can affect both erythropoiesis and megakaryopoiesis [153,154]. Most mutations identified in this gene are missense mutations that primarily affect exon 2 but have been reported in all exons. The result is the loss of negative regulation of the JAK-STAT pathway, which may contribute to the clinical phenotype of erythrocytosis. Multiple studies have found that *LNK* somatic mutations are present in *JAK2* negative MPN cases, with an increased frequency in ET and PMF [55]. *LNK* mutations have also been implicated in idiopathic erythrocytosis and familial MPNs [54,153,155,156]. In an attempt to identify this link between *LNK* mutations and familial cases of *JAK*-negative PV, 94 MPN families were studied by Rumi et al. [156]. Whole-exome sequencing was applied to 16 families; one patient with familial PV was found to be *JAK2* mutation-negative, but *LNK* (E208Q)-positive. When the remaining 93 families were scanned for this mutation using Sanger sequencing, one other patient was also found to harbor this mutation. Germline testing then revealed that both patients carried the *LNK*E208Q mutation, but the family members were negative for this germline mutation. Thus, it is recommended that patients with *JAK2*-V617F and exon 12-negative PV be tested with *LNK* gene sequencing to find any underlying germline causes for erythrocytosis [94,156].

Post-PV and ET myelofibrosis and progression to AML are known consequences of MPNs. TP53 mutations have been identified in over 40% of cases of secondary AMLs; however, missense mutations in this gene are generally found to be early events [46]. TP53 and DNMT3A mutations are more frequently identified in post-PV or post-ET cases [157,158]. Secondary AML have been associated with mutations in *ASXL1*, *SRSF2, IDH1/2*, *CBL,* and *LNK* [46]. *RUNX1* mutations can be found in about 30% of secondary AML cases and are considered a late event [158].

Grinfeld et al. analyzed the mutational profiles of 2035 MPN patients and defined eight genomic subclassification groups. They incorporated driver mutations, cooperating mutations, as well as clinical and laboratory data into the groups in order to create personalized predictions of patient outcomes [159].

## 4. Chronic Neutrophilic Leukemia and CSF3R Mutations

Chronic neutrophilic leukemia (CNL) is defined by the WHO as absolute neutrophilia without left-shift or dysplasia identified [1]. Up to 80–90% of CNL cases harbor mutations in the *CSF3R* gene, which encodes for the granulocyte colony-stimulating factor (G-CSFR) [93]. Thus, this gene functions in neutrophil production and differentiation. As mentioned earlier, G-CSFR is a cytokine receptor that is associated with JAK2. Any mutations in G-CSFR can directly affect the JAK-STAT pathway and subsequent production and differentiation of neutrophils [12,52,93]. Two mutations of clinical significance have been described: T618I, a point mutation in the *CSF3R* gene, located in the membrane region of exon 14, and frameshift or nonsense mutation in exon 17, which results in the truncation of the protein’s cytoplasmic tail [52]. The first mutation is a lot more common and activates the JAK-STAT pathway. JAK2 inhibitor ruxolitinib can be used in these cases with good response [53]. The WHO has included the presence of *CSF3R* mutation as one of the diagnostic requirements to diagnose CNL. Other causes of reactive neutrophilia or myeloid neoplasms must be ruled out before making this diagnosis.

In order to make the diagnosis, *BCR-ABL1* fusion and driver mutations in *JAK2*, *CALR,* and *MPL* must be ruled out. Mutations in *CSF3R* and rearrangements in *PDGFRA*, *PDGFRB*, *FGFR1,* and *PCM1-JAK2* fusion have to be assessed as well. Cooperating mutations are common in CNL as well. Approximately 50–60% cases have *ASXL1* mutations, while 30–40% case harbor *SETBP1* [160,161]. Both mutations have been associated with disease progression and poor survival, and SETBP1 has further been implicated in JAK2 inhibitor resistance [161]. NGS can be used to evaluate the presence of *CSF3R* along with other known mutations such as *ASXL1* and *SETBP1*.

## 5. Chronic Eosinophilic Leukemia

Chronic eosinophilic leukemia, not otherwise specified (CEL, NOS), is a rare clonal proliferation of eosinophils and their precursors, which accumulate in blood and marrow and cause tissue damage secondary to the cellular infiltration and subsequent protein, cytokine, and enzyme release. CEL, NOS does not have a specifically identified driver mutation and therefore is a diagnosis of exclusion, where *BCR-ABL1*, *PCM1-JAK2*, *ETV6-JAK2*, or *BCR-JAK2* fusions are absent, as are *PDGFRA*, *PDGFRB*, and FGFR1 rearrangements [1]. Up to 28% of CEL, NOS patients have been shown to have somatic mutations in myeloid-associated genes including *KIT*, *ASXL1*, *U2AF1*, *EZH2*, *SETBP1*, *ETV6*, and *CSF3R* [162,163,164].

## 6. Summary and Conclusions

**Figure 1:**
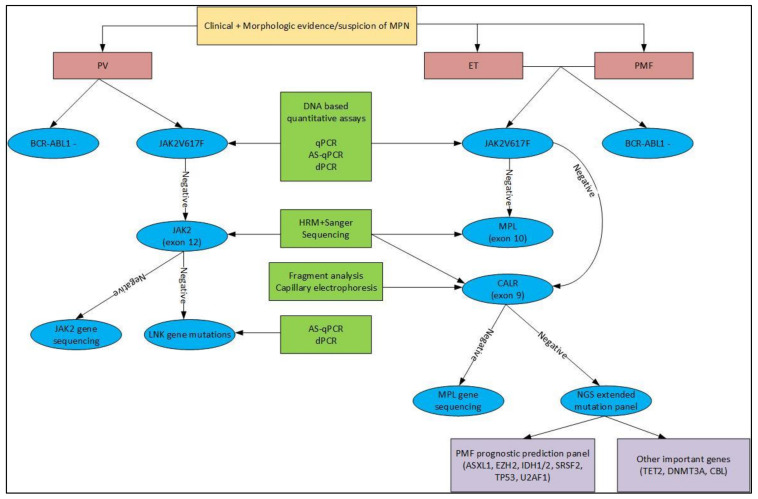
Molecular workup for Philadelphia chromosome-negative myeloproliferative neoplasms.

Due to the rapidly growing field of molecular technology and bioinformatics, our knowledge about disease diagnosis, prognosis, and therapeutic options has increased substantially. Our proposed algorithm for molecular workup of MPNs and commonly used methods are presented in Figure 1. With the easy availability and relatively low cost of sophisticated molecular technologies such as NGS, the diagnostic and prognostic workup of cases has changed dramatically from what it was less than a decade ago. Morphology and is still an important factor in making an initial diagnosis; however, genetic profiling using NGS or whole-genome sequencing has become increasingly important in the diagnosis of challenging cases as well as adding prognostic information. The yield of clinically significant information and actionable mutations has led to the assumption that such methodologies will become increasingly important in routine diagnostics in the future.

This figure shows a proposed diagnostic algorithm for the presence of known driver and non-driver mutations along with the method of detection. When there is a clinical suspicion of MPN with morphologic evidence, the first step is to rule out *BCR-ABL1* translocation. *JAK2* V617F is the most common mutation identified in all three Ph- MPNs and should be identified (or ruled out) using DNA-based quantitative assays. In PV, *JAK2* V617F negative samples can then undergo high-resolution melt analysis (HRM) along with Sanger Sequencing to detect *JAK2* exon 12 mutations. In rare PV cases that are negative for both above mutations, the next step would be to sequence the *JAK2* gene and use allele-specific quantitative PCR (AS-qPCR) or digital PCR (ddPCR) to detect any *LNK* gene mutations. In *JAK2* V617F negative ET and PMF cases, the next step is to identify *MPL* exon 10 and *CALR* exon 9 mutations. HRM with Sanger sequencing can be used for both, while fragment analysis capillary electrophoresis can also be used for *CALR* mutation detection. If both of these are negative as well, the next steps would include *MPL* gene sequencing and NGS with a broad panel of non-driver genes. Alternatively, NGS panels that includes the driver genes along with the non-driver genes can be used as well.

## Figures and Tables

**Table 1:** Most common driver mutations in MPNs.

		Diagnosis				
		CML	PV	ET	PMF	CNL
**Gene**	*BCR-ABL1*	100%	-	-	-	-
	*JAK2*V617F	-	>95%	50–60%	50–60%	-
	*JAK2* exon 12	-	3–4%	-	-	-
	*CALR*	-	-	25%	20–24%	-
	*MPL*	-	-	1–4%	8–10%	-
	*CSFR3*	-	-	-	-	80–90%

**Table 2:** Non-driver genes implicated in *BCR-ABL1* negative Myeloproliferative Neoplasms *.

Gene	Function	Prognostic Implication
***ASXL1***	Histone modification (Chromatin binding protein)	Also identified in age-related clonal hematopoiesis, CML and MPNs—associated with unfavorable prognosis and rapid progression to AML
20q11		
***EZH2***	Histone modification (loss of function of H3K27 methyltransferase)	Implicated in Post-ET and Post-PV MF, unfavorable prognosis in PMF
7q35-36		
***DNMT3A***	DNA methylation (DNA methylase)	Somatic mutations identified in age-related clonal hematopoiesis, CML and MPNs—associated with rapid disease progression
2p23		
***IDH1/2***	DNA methylation (converts isocitrate to α-ketoglutarate)	Implicated in disease progression
2q33.3/15q26.1		
***TET2***	DNA methylation (essential in myelopoiesis)	Somatic mutations identified in age-related clonal hematopoiesis, CML and MPNs—associated with rapid disease progression
4q24		
***SRSF2***	RNA splicing/spliceosome assembly	Associated with progression to Myelofibrosis, downregulates *EZH2,* unfavorable prognosis in PMF
12q25.1		
***SF3B1***	RNA splicing/spliceosome assembly	Associated with the presence of ringed sideroblasts, increased incidence of anemia
2q33.1		
***U2AF1***	RNA splicing/spliceosome assembly	Associated with disease progression to AML, unfavorable prognosis in PMF
21q22.3		
***LNK***	Negative regulator of JAK2	Implicated in disease progression, also found in familial cases of erythrocytosis
12q24		
***TP53***	Transcription factor (involved in cell cycle regulation, DNA repair and apoptosis)	TP53 associated with poor prognosis, also associated with disease progression to AML (in <3% cases)
17p13.1		
***ETV6***	Transcription factor	Also associated with disease progression to AML (in <3% cases)
12p13		
***RUNX1***	Transcription factor (hematopoiesis)	Also associated with disease progression to AML (in <3% cases), also seen in secondary AML (about 30% cases)
21q22.3		
***CBL***	Cytokine receptor in signal transduction pathways	Implicated in AML progression (rare in PMF, but found in 10–15% secondary AML)
11q23.3		
***FLT3***	Cytokine receptor in signal transduction pathways	Implicated in AML progression (rare in PMF, but found in 10–15% secondary AML)
13q12		
***NRAS***	MAPK signaling pathway	Implicated in AML progression (rare in PMF, but found in 10–15% secondary AML)
1p13.2		
***NF1***	MAPK signaling pathway	Implicated in AML progression (rare in PMF, but found in 10–15% secondary AML)
17q11		

* Data adapted from Rumi and Cazzola (2017) and Vainchenker and Kralovics (2017) [5,46].

## References

[1] Swerdlow S.H., Campo E., Harris N.L., Jaffe E.S., Pileri S.A., Stein H., Thiele J. (2017). WHO Classification of Tumours of Haematopoietic and Lymphoid Tissues.

[2] Dameshek W. (1951). Editorial: Some Speculations on the Myeloproliferative Syndromes. Blood.

[3] Rowley J.D. (1973). A New Consistent Chromosomal Abnormality in Chronic Myelogenous Leukaemia identified by Quinacrine Fluorescence and Giemsa Staining. Nature.

[4] Nowell P., Hungerford D. (1960). A minute chromosome in human chronic granulocytic leukemia. Science.

[5] Rumi E., Cazzola M. (2017). Diagnosis, risk stratification, and response evaluation in classical myeloproliferative neoplasms. Blood.

[6] James C., Ugo V., Le Couédic J.-P., Staerk J., Delhommeau F., Lacout C., Garçon L., Raslova H., Berger R., Griscelli A.B. (2005). A unique clonal JAK2 mutation leading to constitutive signalling causes polycythaemia vera. Nature.

[7] Kralovics R., Passamonti F., Buser A.S., Teo S.-S., Tiedt R., Passweg J.R., Tichelli A., Cazzola M., Skoda R.C. (2005). A Gain-of-Function Mutation ofJAK2in Myeloproliferative Disorders. N. Engl. J. Med..

[8] Klampfl T., Gisslinger H., Harutyunyan A., Nivarthi H., Rumi E., Milosevic J.D., Them N.C., Berg T., Gisslinger B., Pietra D. (2013). Somatic Mutations of Calreticulin in Myeloproliferative Neoplasms. N. Engl. J. Med..

[9] Nangalia J., Massie C.E., Baxter E.J., Nice F.L., Gundem G., Wedge D.C., Avezov E., Li J., Kollmann K., Kent D.G. (2013). Somatic CALR Mutations in Myeloproliferative Neoplasms with Nonmutated JAK2. N. Engl. J. Med..

[10] Pikman Y., Lee B.H., Mercher T., McDowell E., Ebert B.L., Gozo M., Cuker A., Wernig G., Moore S., Galinsky I. (2006). MPLW515L Is a Novel Somatic Activating Mutation in Myelofibrosis with Myeloid Metaplasia. PLoS Med..

[11] Scott L., Tong W., Levine R.L., Scott M.A., Beer P.A., Stratton M.R., Futreal P.A., Erber W., McMullin M.F., Harrison C.N. (2007). JAK2Exon 12 Mutations in Polycythemia Vera and Idiopathic Erythrocytosis. N. Engl. J. Med..

[12] Westermann J., Bullinger L. (2021). Precision medicine in myeloid malignancies. Semin. Cancer Biol..

[13] Ghalesardi O.K., Khosravi A., Azizi E., Ahmadi S.E., Hajifathali A., Bonakchi H., Shahidi M. (2021). The prognostic importance of BCR-ABL transcripts in Chronic Myeloid Leukemia: A systematic review and meta-analysis. Leuk. Res..

[14] Bavaro L., Martelli M., Cavo M., Soverini S. (2019). Mechanisms of Disease Progression and Resistance to Tyrosine Kinase Inhibitor Therapy in Chronic Myeloid Leukemia: An Update. Int. J. Mol. Sci..

[15] Gong Z., Medeiros L.J., Cortes J., Zheng L., Khoury J.D., Wang W., Tang G., Loghavi S., Luthra R., Yang W. (2017). Clinical and prognostic significance of e1a2 BCR-ABL1 transcript subtype in chronic myeloid leukemia. Blood Cancer J..

[16] Burmeister T., Reinhardt R. (2008). A multiplex PCR for improved detection of typical and atypical BCR-ABL fusion transcripts. Leuk. Res..

[17] Bennour A., Ouahchi I., Moez M., Elloumi M., Khelif A., Saad A., Sennana H. (2012). Comprehensive analysis of BCR/ABL variants in chronic myeloid leukemia patients using multiplex RT-PCR. Clin. Lab..

[18] Jain P., Kantarjian H., Patel K.P., Gonzalez G.N., Luthra R., Kanagal-Shamanna R., Sasaki K., Jabbour E., Romo C.G., Kadia T.M. (2016). Impact of BCR-ABL transcript type on outcome in patients with chronic-phase CML treated with tyrosine kinase inhibitors. Blood.

[19] Perego R., Costantini M., Cornacchini G., Gargantini L., Bianchi C., Pungolino E., Rovida E., Morra E. (2000). The possible influences of B2A2 and B3A2 BCR/ABL protein structure on thrombopoiesis in chronic myeloid leukaemia. Eur. J. Cancer.

[20] Hanfstein B., Lauseker M., Hehlmann R., Saussele S., Erben P., Dietz C., Fabarius A., Proetel U., Schnittger S., Haferlach C. (2014). Distinct characteristics of e13a2 versus e14a2 BCR-ABL1 driven chronic myeloid leukemia under first-line therapy with imatinib. Haematologica.

[21] Hur M., Song E., Kang S.H., Shin D.H., Kim J., Park S., Cho H. (2002). Lymphoid preponderance and the absence of basophilia and splenomegaly are frequent in m-bcr-positive chronic myelogenous leukemia. Ann. Hematol..

[22] Ohtake S. (2002). Chronic Myelogenous Leukemia with p190BCR-ABL Expression: The Missing Link with Monocytosis. Intern. Med..

[23] Van Rhee F., Hochhaus A., Lin F., Melo J., Goldman J., Cross N. (1996). p190 BCR-ABL mRNA is expressed at low levels in p210-positive chronic myeloid and acute lymphoblastic leukemias. Blood.

[24] Baccarani M., Castagnetti F., Gugliotta G., Rosti G., Soverini S., Albeer A., Pfirrmann M., for the International BCR-ABL Study Group (2019). The proportion of different BCR-ABL1 transcript types in chronic myeloid leukemia. An international overview. Leukemia.

[25] Arun A.K., Senthamizhselvi A., Mani S., Vinodhini K., Janet N.B., Lakshmi K.M., Abraham A., George B., Srivastava A., Srivastava V.M. (2017). Frequency of rare BCR-ABL1 fusion transcripts in chronic myeloid leukemia patients. Int. J. Lab. Hematol..

[26] Catania G., Monaco F., Pini M., Corsetti M.T., Salvio M., Trincheri N., Zallio F., Pietrasanta D., Baraldi A., Lorella D. (2014). Prognostic Impact of p190 and p210 Co-Expression at Diagnosis in Chronic Myeloid Leukemia (CML) Patients Treated with Imatinib. Blood.

[27] Castagnetti F., Gugliotta G., Palandri F., Breccia M., Specchia G., Abruzzese E., Intermesoli T., Capucci A., Martino B., Stagno F. (2010). BCR-ABL Fusion Transcript Do Not Significantly Influence the Outcome of Chronic Myeloid Leukemia Patients in Early Chronic Phase Treated with Imatinib Mesylate: A GIMEMA CML WP Analysis. Blood.

[28] Vega-Ruiz A., Kantarjian H., Shan J., Wierda W., Burger J., Verstovsek S., Garcia-Manero G., Cortes J. (2007). Better Molecular Response to Imatinib for Patients (pts) with Chronic Myeloid Leukemia (CML) in Chronic Phase (CP) Carrying the b3a2 Transcript Compared to b2a2. Blood.

[29] Erbilgin Y., Eskazan A.E., Ng O.H., Salihoglu A., Elverdi T., Firtina S., Tasar O., Mercan S., Sisko S., Khodzhaev K. (2019). Deep sequencing of BCR-ABL1 kinase domain mutations in chronic myeloid leukemia patients with resistance to tyrosine kinase inhibitors. Leuk. Lymphoma.

[30] Cumbo C., Anelli L., Specchia G., Albano F. (2020). Monitoring of Minimal Residual Disease (MRD) in Chronic Myeloid Leukemia: Recent Advances. Cancer Manag. Res..

[31] Hochhaus A., Baccarani M., Silver R.T., Schiffer C., Apperley J.F., Cervantes F., Clark R.E., Cortes J.E., Deininger M.W., Guilhot F. (2020). European LeukemiaNet 2020 recommendations for treating chronic myeloid leukemia. Leukemia.

[32] Kizilors A., Crisà E., Lea N., Passera R., Mian S., Anwar J., Best S., Nicolini F.E., Ireland R., Aldouri M. (2019). Effect of low-level BCR-ABL1 kinase domain mutations identified by next-generation sequencing in patients with chronic myeloid leukaemia: A population-based study. Lancet Haematol..

[33] Liu J., Yang H., Xu X., Yi S., Meng L. (2020). Mutations in the BCR-ABL1 kinase domain in patients with chronic myeloid leukaemia treated with TKIs or at diagnosis. Oncol. Lett..

[34] Soverini S., De Benedittis C., Polakova K.M., Linhartova J., Castagnetti F., Gugliotta G., Papayannidis C., Mancini M., Klamová H., Salvucci M. (2016). Next-generation sequencing for sensitive detection of BCR-ABL1 mutations relevant to tyrosine kinase inhibitor choice in imatinib-resistant patients. Oncotarget.

[35] Branford S. (2016). Molecular monitoring in chronic myeloid leukemia—How low can you go?. Hematology.

[36] Schnittger S., Haferlach C., Nadarajah M.N., Meggendorfer M., Fasan A., Rose D., Alpermann T., Kern W., Haferlach T. (2014). CML Patients with Resistance to Tyrosine Kinase Inhibitors and without BCR-ABL1 Resistance Mutation Frequently Carry Other Gene Mutations. Blood.

[37] Imataki O., Ishida T., Kubo H., Uemura M., Nanya Y., Kawakami K., Ogawa S., Kadowaki N. (2020). A Case of Tyrosine Kinase Inhibitor-Resistant Chronic Myeloid Leukemia, Chronic Phase with ASXL1 Mutation. Case Rep. Oncol..

[38] Deininger M.W., Shah N.P., Altman J.K., Berman E., Bhatia R., Bhatnagar B., DeAngelo D.J., Gotlib J., Hobbs G., Maness L. (2020). Chronic Myeloid Leukemia, Version 2.2021, NCCN Clinical Practice Guidelines in Oncology. J. Natl. Compr. Cancer Netw..

[39] Hehlmann R. (2020). The new European leukemianet recommendations for treating CML. Hematol. Transfus. Cell Ther..

[40] Rack K.A., Van Den Berg E., Haferlach C., Beverloo H.B., Costa D., Espinet B., Foot N., Jeffries S., Martin K., O’Connor S. (2019). European recommendations and quality assurance for cytogenomic analysis of haematological neoplasms. Leukemia.

[41] Shanmuganathan N., Hughes T.P. (2018). Molecular monitoring in CML: How deep? How often? How should it influence therapy?. Blood.

[42] Guérin A., Chen L., Dea K., Wu E.Q., Goldberg S.L. (2014). Association between regular molecular monitoring and tyrosine kinase inhibitor therapy adherence in chronic myelogenous leukemia in the chronic phase. Curr. Med. Res. Opin..

[43] Jabbour E., Kantarjian H.M., Saglio G., Steegmann J.L., Shah N.P., Boqué C., Chuah C., Pavlovsky C., Mayer J., Cortes J. (2014). Early response with dasatinib or imatinib in chronic myeloid leukemia: 3-year follow-up from a randomized phase 3 trial (DASISION). Blood.

[44] O’Hare T., Eide C.A., Deininger M.W.N. (2007). Bcr-Abl kinase domain mutations, drug resistance, and the road to a cure for chronic myeloid leukemia. Blood.

[45] How J., Hobbs G.S., Mullally A. (2019). Mutant calreticulin in myeloproliferative neoplasms. Blood.

[46] Vainchenker W., Kralovics R. (2017). Genetic basis and molecular pathophysiology of classical myeloproliferative neoplasms. Blood.

[47] Jang M.-A., Choi C.W. (2020). Recent insights regarding the molecular basis of myeloproliferative neoplasms. Korean J. Intern. Med..

[48] Stuckey R., Gómez-Casares M.T. (2021). Recent advances in the use of molecular analyses to inform the diagnosis and prognosis of patients with polycythaemia vera. Int. J. Mol. Sci..

[49] Guglielmelli P., Nangalia J., Green A.R., Vannucchi A.M. (2014). CALR mutations in myeloproliferative neoplasms: Hidden behind the reticulum. Am. J. Hematol..

[50] Vantyghem S., Peterlin P., Thépot S., Ménard A., Dubruille V., Debord C., Guillaume T., Garnier A., Le Bourgeois A., Wuilleme S. (2021). Diagnosis and prognosis are supported by integrated assessment of next-generation sequencing in chronic myeloid malignancies. A real-life study. Haematologica.

[51] Cazzola M., Kralovics R. (2014). From Janus kinase 2 to calreticulin: The clinically relevant genomic landscape of myeloproliferative neoplasms. Blood.

[52] Maxson J.E., Gotlib J., Pollyea D.A., Fleischman A.G., Agarwal A., Eide C.A., Bottomly D., Wilmot B., McWeeney S.K., Tognon C.E. (2013). Oncogenic CSF3R Mutations in Chronic Neutrophilic Leukemia and Atypical CML. N. Engl. J. Med..

[53] Pardanani A., Lasho T.L., Laborde R.R., Elliott M., Hanson C.A., Knudson R.A., Ketterling R.P., Maxson J.E., Tyner J.W., Tefferi A. (2013). CSF3R T618I is a highly prevalent and specific mutation in chronic neutrophilic leukemia. Leukemia.

[54] Lasho T.L., Pardanani A., Tefferi A. (2010). LNK Mutations in JAK2 Mutation–Negative Erythrocytosis. N. Engl. J. Med..

[55] Oh S.T., Simonds E.F., Jones C., Hale M.B., Goltsev Y., Gibbs K.D., Merker J.D., Zehnder J.L., Nolan G.P., Gotlib J. (2010). Novel mutations in the inhibitory adaptor protein LNK drive JAK-STAT signaling in patients with myeloproliferative neoplasms. Blood.

[56] Schwaab J., Ernst T., Erben P., Rinke J., Schnittger S., Ströbel P., Metzgeroth G., Mossner M., Haferlach T., Cross N.C.P. (2012). Activating CBL mutations are associated with a distinct MDS/MPN phenotype. Ann. Hematol..

[57] Plo I., Bellanné-Chantelot C., Mosca M., Mazzi S., Marty C., Vainchenker W. (2017). Genetic Alterations of the Thrombopoietin/MPL/JAK2 Axis Impacting Megakaryopoiesis. Front. Endocrinol..

[58] Ross D.M., Thomson C., Hamad N., Lane S.W., Manos K., Grigg A.P., Guo B., Erber W.N., Scott A., Viiala N. (2021). Myeloid somatic mutation panel testing in myeloproliferative neoplasms. Pathology.

[59] Accurso V., Santoro M., Mancuso S., Napolitano M., Carlisi M., Mattana M., Russo C., Di Stefano A., Sirocchi D., Siragusa S. (2020). The Essential Thrombocythemia in 2020: What We Know and Where We Still Have to Dig Deep. Clin. Med. Insights Blood Disord..

[60] Angona A., Fernández-Rodríguez C., Álvarez-Larrán A., Camacho L., Longarón R., Torres E., Pairet S., Besses C., Bellosillo B. (2016). Molecular characterisation of triple negative essential thrombocythaemia patients by platelet analysis and targeted sequencing. Blood Cancer J..

[61] Tefferi A. (2021). Primary myelofibrosis: 2021 update on diagnosis, risk-stratification and management. Am. J. Hematol..

[62] Tefferi A., Guglielmelli P., Pardanani A., Vannucchi A.M. (2018). Myelofibrosis Treatment Algorithm 2018. Blood Cancer J..

[63] Palumbo G.A., Stella S., Pennisi M.S., Pirosa C., Fermo E., Fabris S., Cattaneo D., Iurlo A. (2019). The Role of New Technologies in Myeloproliferative Neoplasms. Front. Oncol..

[64] Grimwade L.F., Happerfield L., Tristram C., McIntosh G., Rees M., Bench A.J., Boyd E.M., Hall M., Quinn A., Piggott N. (2009). Phospho-STAT5 and phospho-Akt expression in chronic myeloproliferative neoplasms. Br. J. Haematol..

[65] Tiedt R., Hao-Shen H., Sobas M.A., Looser R., Dirnhofer S., Schwaller J., Skoda R.C. (2008). Ratio of mutant JAK2-V617F to wild-type Jak2 determines the MPD phenotypes in transgenic mice. Blood.

[66] Szpurka H., Tiu R., Murugesan G., Aboudola S., Hsi E.D., Theil K.S., Sekeres M.A., Maciejewski J.P. (2006). Refractory anemia with ringed sideroblasts associated with marked thrombocytosis (RARS-T), another myeloproliferative condition characterized by JAK2 V617F mutation. Blood.

[67] Hirsch P., Mamez A.C., Belhocine R., Lapusan S., Tang R., Suner L., Bories D., Marzac C., Fava F., Legrand O. (2016). Clonal history of a cord blood donor cell leukemia with prenatal somatic JAK2 V617F mutation. Leukemia.

[68] McKerrell T., Park N., Moreno T., Grove C.S., Ponstingl H., Stephens J., Crawley C., Craig J., Scott M.A., Hodkinson C. (2015). Leukemia-Associated Somatic Mutations Drive Distinct Patterns of Age-Related Clonal Hemopoiesis. Cell Rep..

[69] Borowczyk M., Wojtaszewska M., Lewandowski K., Gil L., Lewandowska M., Lehmann-Kopydłowska A., Kroll-Balcerzak R., Balcerzak A., Iwoła M., Michalak M. (2015). The JAK2 V617F mutational status and allele burden may be related with the risk of venous thromboembolic events in patients with Philadelphia-negative myeloproliferative neoplasms. Thromb. Res..

[70] Mesa R.A. (2017). New Guidelines from the NCCN for Polycythemia Vera. Clin. Adv. Hematol. Oncol..

[71] Passamonti F., Rumi E., Pietra D., Elena C., Boveri E., Arcaini L., Roncoroni E., Astori C., Merli M., Boggi S. (2010). A prospective study of 338 patients with polycythemia vera: The impact of JAK2 (V617F) allele burden and leukocytosis on fibrotic or leukemic disease transformation and vascular complications. Leukemia.

[72] Zhou F.-P., Wang C.-C., Du H.-P., Cao S.-B., Zhang J. (2020). Primary myelofibrosis with concurrent CALR and MPL mutations: A case report. World J. Clin. Cases.

[73] McGaffin G., Harper K., Stirling D., McLintock L. (2014). JAK2 V617F and CALR mutations are not mutually exclusive; findings from retrospective analysis of a small patient cohort. Br. J. Haematol..

[74] Rashid M., Ahmed R.Z., Ahmed S., Nadeem M., Ahmed N., Shamsi T.S. (2016). Coexisting JAK2V617F and CALR Exon 9 Mutation in Essential Thrombocythemia. Indian J. Hematol. Blood Transfus..

[75] Hussein K., Theophile K., Buhr T., Beller A., Kreipe H., Bock O. (2009). Different lineage involvement in myelodysplastic/myeloproliferative disease with combined MPLW515L and JAK2V617F mutation: Correspondence. Br. J. Haematol..

[76] Tefferi A., Lasho T.L., Finke C.M., Knudson R.A., Ketterling R., Hanson C.H., Maffioli M., Caramazza D., Passamonti F., Pardanani A. (2014). CALR vs JAK2 vs MPL-mutated or triple-negative myelofibrosis: Clinical, cytogenetic and molecular comparisons. Leukemia.

[77] Loscocco G.G., Guglielmelli P., Vannucchi A.M. (2020). Impact of Mutational Profile on the Management of Myeloproliferative Neoplasms: A Short Review of the Emerging Data. OncoTargets Ther..

[78] Rumi E., Pietra D., Pascutto C., Guglielmelli P., Martínez-Trillos A., Casetti I., Colomer D., Pieri L., Pratcorona M., Rotunno G. (2014). Clinical effect of driver mutations of JAK2, CALR, or MPL in primary myelofibrosis. Blood.

[79] Rumi E., Pietra D., Ferretti V.V., Klampfl T., Harutyunyan A., Milosevic J.D., Them N.C.C., Berg T., Elena C., Casetti I.C. (2014). JAK2 or CALR mutation status defines subtypes of essential thrombocythemia with substantially different clinical course and outcomes. Blood.

[80] Tefferi A., Guglielmelli P., Larson D.R., Finke C., Wassie E.A., Pieri L., Gangat N., Fjerza R., Belachew A.A., Lasho T.L. (2014). Long-term survival and blast transformation in molecularly annotated essential thrombocythemia, polycythemia vera, and myelofibrosis. Blood.

[81] Tefferi A., Barbui T. (2020). Polycythemia vera and essential thrombocythemia: 2021 update on diagnosis, risk-stratification and management. Am. J. Hematol..

[82] Patterson-Fortin J., Moliterno A.R. (2017). Molecular Pathogenesis of Myeloproliferative Neoplasms: Influence of Age and Gender. Curr. Hematol. Malig. Rep..

[83] Defour J.-P., Chachoua I., Pecquet C., Constantinescu S. (2016). Oncogenic activation of MPL/thrombopoietin receptor by 17 mutations at W515: Implications for myeloproliferative neoplasms. Leukemia.

[84] Beer P.A., Campbell P.J., Scott L.M., Bench A.J., Erber W.N., Bareford D., Wilkins B.S., Reilly J.T., Hasselbalch H.C., Bowman R. (2008). MPL mutations in myeloproliferative disorders: Analysis of the PT-1 cohort. Blood.

[85] Malcovati L., Della Porta M.G., Pietra D., Boveri E., Pellagatti A., Galli’ A., Travaglino E., Brisci A., Rumi E., Passamonti F. (2009). Molecular and clinical features of refractory anemia with ringed sideroblasts associated with marked thrombocytosis. Blood.

[86] Cabagnols X., Favale F., Pasquier F., Messaoudi K., Defour J.-P., Ianotto J.C., Marzac C., Le Couédic J.P., Droin N., Chachoua I. (2016). Presence of atypical thrombopoietin receptor (MPL) mutations in triple-negative essential thrombocythemia patients. Blood.

[87] Feenstra J.D.M., Nivarthi H., Gisslinger H., Leroy E., Rumi E., Chachoua I., Bagienski K., Kubesova B., Pietra D., Gisslinger B. (2016). Whole-exome sequencing identifies novel MPL and JAK2 mutations in triple-negative myeloproliferative neoplasms. Blood.

[88] Ding J., Komatsu H., Wakita A., Kato-Uranishi M., Ito M., Satoh A., Tsuboi K., Nitta M., Miyazaki H., Iida S. (2004). Familial essential thrombocythemia associated with a dominant-positive activating mutation of the c-MPL gene, which encodes for the receptor for thrombopoietin. Blood.

[89] Dasouki M.J., Rafi S.K., Olm-Shipman A.J., Wilson N.R., Abhyankar S., Ganter B., Furness L.M., Fang J., Calado R.T., Saadi I. (2013). Exome sequencing reveals a thrombopoietin ligand mutation in a Micronesian family with autosomal recessive aplastic anemia. Blood.

[90] Oudenrijn S.V.D., Bruin M., Folman C.C., Peters M., Faulkner L.B., De Haas M., Borne A.E.G.K.V.D. (2000). Mutations in the thrombopoietin receptor, Mpl, in children with congenital amegakaryocytic thrombocytopenia. Br. J. Haematol..

[91] Germeshausen M., Ballmaier M., Welte K. (2006). MPL mutations in 23 patients suffering from congenital amegakaryocytic thrombocytopenia: The type of mutation predicts the course of the disease. Hum. Mutat..

[92] Ghilardi N., Wiestner A., Kikuchi M., Ohsaka A., Skoda R.C. (1999). Hereditary thrombocythaemia in a Japanese family is caused by a novel point mutation in the thrombopoietin gene. Br. J. Haematol..

[93] Zuo Z., Li S., Xu J., You M.J., Khoury J.D., Yin C.C. (2019). Philadelphia-Negative Myeloproliferative Neoplasms: Laboratory Workup in the Era of Next-Generation Sequencing. Curr. Hematol. Malig. Rep..

[94] Guglielmelli P., Pietra D., Pane F., Pancrazzi A., Cazzola M., Vannucchi A.M., Tura S., Barosi G. (2017). Recommendations for molecular testing in classical Ph1-neg myeloproliferative disorders—A consensus project of the Italian Society of Hematology. Leuk. Res..

[95] Lippert E., Girodon F., Hammond E., Jelinek J., Reading N.S., Fehse B., Hanlon K., Hermans M., Richard C., Swierczek S. (2009). Concordance of assays designed for the quantification of JAK2V617F: A multicenter study. Haematologica.

[96] Merker J.D., Jones C.D., Oh S.T., Schrijver I., Gotlib J., Zehnder J.L. (2010). Design and Evaluation of a Real-Time PCR Assay for Quantification of JAK2 V617F and Wild-Type JAK2 Transcript Levels in the Clinical Laboratory. J. Mol. Diagn..

[97] Jovanovic J.V., Ivey A., Vannucchi A.M., Lippert E., Leibundgut E.O., Cassinat B., Pallisgaard N., Maroc N., Hermouet S., Nickless G. (2013). Establishing optimal quantitative-polymerase chain reaction assays for routine diagnosis and tracking of minimal residual disease in JAK2-V617F-associated myeloproliferative neoplasms: A joint European LeukemiaNet/MPN&MPNr-EuroNet (COST action BM0902) study. Leukemia.

[98] Waterhouse M., Follo M., Pfeifer D., Von Bubnoff N., Duyster J., Bertz H., Finke J. (2016). Sensitive and accurate quantification of JAK2 V617F mutation in chronic myeloproliferative neoplasms by droplet digital PCR. Ann. Hematol..

[99] Yow K.S., Liu X., Chai C.N., Tung M.L., Yan B., Christopher D., Ong K.H., Ooi M.G. (2020). Relationship of JAK2 (V617F) Allelic Burden with Clinico- Haematological Manifestations of Philadelphia-Negative Myeloproliferative Neoplasms. Asian Pac. J. Cancer Prev..

[100] Salmoiraghi S., Belotti C., Finazzi M.C., Mico M.C., Algarotti A., Finazzi G., Mascheroni M., Salvi A., Rambaldi A., Spinelli O. (2017). Minimal residual disease monitoring by digital PCR for JAK2V617F detection in patients with myelofibrosis (MF) or acute myeloid leukemia secondary to MF after allogeneic stem cell transplantation. Haematologica.

[101] Sidon P., El Housni H., Dessars B., Heimann P. (2006). The JAK2V617F mutation is detectable at very low level in peripheral blood of healthy donors. Leukemia.

[102] Klco J., Vij R., Kreisel F.H., Hassan A., Frater J.L. (2010). Molecular Pathology of Myeloproliferative Neoplasms. Am. J. Clin. Pathol..

[103] Passamonti F., Elena C., Schnittger S., Skoda R.C., Green A.R., Girodon F., Kiladjian J.-J., McMullin M.F., Ruggeri M., Besses C. (2011). Molecular and clinical features of the myeloproliferative neoplasm associated with JAK2 exon 12 mutations. Blood.

[104] Furtado L.V., Weigelin H.C., Elenitoba-Johnson K.S., Betz B.L. (2013). A Multiplexed Fragment Analysis-Based Assay for Detection of JAK2 Exon 12 Mutations. J. Mol. Diagn..

[105] Carillo S., Henry L., Lippert E., Girodon F., Guiraud I., Richard C., Galopin F.D., Cleyrat C., Jourdan E., Kralovics R. (2011). Nested High-Resolution Melting Curve Analysis: A Highly Sensitive, Reliable, and Simple Method for Detection of Jak2 Exon 12 Mutations—Clinical Relevance in the Monitoring of Polycythemia. J. Mol. Diagn..

[106] Jones A.V., Ward D., Lyon M., Leung W., Callaway A., Chase A., Dent C.L., White H.E., Drexler H.G., Nangalia J. (2015). Evaluation of methods to detect CALR mutations in myeloproliferative neoplasms. Leuk. Res..

[107] Rotunno G., Mannarelli C., Guglielmelli P., Pacilli A., Pancrazzi A., Pieri L., Fanelli T., Bosi A., Vannucchi A.M. (2014). Impact of calreticulin mutations on clinical and hematological phenotype and outcome in essential thrombocythemia. Blood.

[108] Szuber N., Lamontagne B., Busque L. (2016). Novel germline mutations in the calreticulin gene: Implications for the diagnosis of myeloproliferative neoplasms. J. Clin. Pathol..

[109] Chi J., Manoloukos M., Pierides C., Nicolaidou V., Nicolaou K., Kleopa M., Vassiliou G., Costeas P. (2015). Calreticulin mutations in myeloproliferative neoplasms and new methodology for their detection and monitoring. Ann. Hematol..

[110] Pietra D., Brisci A., Rumi E., Boggi S., Elena C., Pietrelli A., Bordoni R., Ferrari M., Passamonti F., De Bellis G. (2011). Deep sequencing reveals double mutations in cis of MPL exon 10 in myeloproliferative neoplasms. Haematologica.

[111] Ghaderi M., Strömberg O., Porwit A. (2010). Rapid real-time PCR assay for detection of MPL W515L mutation in patients with chronic myeloproliferative disorders. Int. J. Lab. Hematol..

[112] Ivanova M.I., Shivarov V.S., Hadjiev E.A., Naumova E.J. (2011). Novel multiplex bead-based assay with LNA-modified probes for detection of MPL exon 10 mutations. Leuk. Res..

[113] Zhuge J., Zhang W., Xu M., Hoffman R., Zhang W. (2010). Sensitive detection of MPLW515L/K mutations by amplification refractory mutation system (ARMS)-PCR. Clin. Chim. Acta.

[114] Boyd E.M., Bench A.J., Goday-Fernández A., Anand S., Vaghela K.J., Beer P., Scott M.A., Bareford D., Green A.R., Huntly B. (2010). Clinical utility of routine MPL exon 10 analysis in the diagnosis of essential thrombocythaemia and primary myelofibrosis: Research paper. Br. J. Haematol..

[115] Schnittger S., Bacher U., Haferlach C., Beelen D., Bojko P., Bürkle D., Dengler R., Distelrath A., Eckart M., Eckert R. (2009). Characterization of 35 new cases with four different MPLW515 mutations and essential thrombocytosis or primary myelofibrosis. Haematologica.

[116] Serratì S., De Summa S., Pilato B., Petriella D., Lacalamita R., Tommasi S., Pinto R. (2016). Next-generation sequencing: Advances and applications in cancer diagnosis. OncoTargets Ther..

[117] Tefferi A., Lasho T.L., Finke C.M., Elala Y., Hanson C.A., Ketterling R.P., Gangat N., Pardanani A. (2016). Targeted deep sequencing in primary myelofibrosis. Blood Adv..

[118] Tefferi A., Lasho T.L., Guglielmelli P., Finke C.M., Rotunno G., Elala Y., Pacilli A., Hanson C.A., Pancrazzi A., Ketterling R.P. (2016). Targeted deep sequencing in polycythemia vera and essential thrombocythemia. Blood Adv..

[119] Steensma D.P., Bejar R., Jaiswal S., Lindsley R.C., Sekeres M., Hasserjian R.P., Ebert B.L. (2015). Clonal hematopoiesis of indeterminate potential and its distinction from myelodysplastic syndromes. Blood.

[120] Delic S., Rose M., Kern W., Nadarajah N., Haferlach C., Haferlach T., Meggendorfer M. (2016). Application of an NGS-based 28-gene panel in myeloproliferative neoplasms reveals distinct mutation patterns in essential thrombocythaemia, primary myelofibrosis and polycythaemia vera. Br. J. Haematol..

[121] Lundberg P., Karow A., Nienhold R., Looser R., Hao-Shen H., Nissen I., Girsberger S., Lehmann T., Passweg J.R., Stern M. (2014). Clonal evolution and clinical correlates of somatic mutations in myeloproliferative neoplasms. Blood.

[122] Ortmann C.A., Kent D.G., Nangalia J., Silber Y., Wedge D.C., Grinfeld J., Baxter E.J., Massie C.E., Papaemmanuil E., Menon S. (2015). Effect of Mutation Order on Myeloproliferative Neoplasms. N. Engl. J. Med..

[123] Zhang X., Su J., Jeong M., Ko M., Huang Y., Park H.J., Guzman A., Lei Y., Huang Y.-H., Rao A. (2016). DNMT3A and TET2 compete and cooperate to repress lineage-specific transcription factors in hematopoietic stem cells. Nat. Genet..

[124] Tefferi A., Pardanani A., Lim K.-H., Abdel-Wahab O., Lasho T.L., Patel J., Gangat N., Finke C.M., Schwager S., Mullally A. (2009). TET2 mutations and their clinical correlates in polycythemia vera, essential thrombocythemia and myelofibrosis. Leukemia.

[125] Cerquozzi S., Barraco D., Lasho T., Finke C., Hanson C.A., Ketterling R.P., Pardanani A., Gangat N., Tefferi A. (2017). Risk factors for arterial versus venous thrombosis in polycythemia vera: A single center experience in 587 patients. Blood Cancer J..

[126] Stegelmann F., Bullinger L., Schlenk R.F., Paschka P., Griesshammer M., Blersch C., Kuhn S., Schauer S.G., Dohner H., Dohner K. (2011). DNMT3A mutations in myeloproliferative neoplasms. Leukemia.

[127] Jacquelin S., Straube J., Cooper L., Vu T., Song A., Bywater M., Baxter E., Heidecker M., Wackrow B., Porter A. (2018). Jak2V617F and Dnmt3a loss cooperate to induce myelofibrosis through activated enhancer-driven inflammation. Blood.

[128] Nangalia J., Nice F.L., Wedge D., Godfrey A.L., Grinfeld J., Thakker C., Massie C., Baxter J., Sewell D., Silber Y. (2015). DNMT3A mutations occur early or late in patients with myeloproliferative neoplasms and mutation order influences phenotype. Haematologica.

[129] Tefferi A., Jimma T., Sulai N.H., Lasho T.L., Finke C.M., Knudson R.A., McClure R.F., Pardanani A. (2011). IDH mutations in primary myelofibrosis predict leukemic transformation and shortened survival: Clinical evidence for leukemogenic collaboration with JAK2V617F. Leukemia.

[130] Tefferi A., Lasho T.L., Abdel-Wahab O., Guglielmelli P., Patel J., Caramazza D., Pieri L., Finke C.M., Kilpivaara O., Wadleigh M. (2010). IDH1 and IDH2 mutation studies in 1473 patients with chronic-, fibrotic- or blast-phase essential thrombocythemia, polycythemia vera or myelofibrosis. Leukemia.

[131] Gross S., Cairns R.A., Minden M.D., Driggers E.M., Bittinger M.A., Jang H.G., Sasaki M., Jin S., Schenkein D.P., Su S.M. (2010). Cancer-associated metabolite 2-hydroxyglutarate accumulates in acute myelogenous leukemia with isocitrate dehydrogenase 1 and 2 mutations. J. Exp. Med..

[132] Yonal-Hindilerden I., Daglar-Aday A., Hindilerden F., Akadam-Teker B., Yilmaz C., Nalcaci M., Yavuz A.S., Sargin D. (2016). The Clinical Significance of IDH Mutations in Essential Thrombocythemia and Primary Myelofibrosis. J. Clin. Med. Res..

[133] Asada S., Kitamura T. (2019). Aberrant histone modifications induced by mutant ASXL1 in myeloid neoplasms. Int. J. Hematol..

[134] Fujino T., Kitamura T. (2020). ASXL1 mutation in clonal hematopoiesis. Exp. Hematol..

[135] Ernst T., Chase A.J., Score J., Hidalgo-Curtis C.E., Bryant C., Jones A.V., Waghorn K., Zoi K., Ross F.M., Reiter A. (2010). Inactivating mutations of the histone methyltransferase gene EZH2 in myeloid disorders. Nat. Genet..

[136] Puda A., Milosevic J.D., Berg T., Klampfl T., Harutyunyan A., Gisslinger B., Rumi E., Pietra D., Malcovati L., Elena C. (2012). Frequent deletions of JARID2 in leukemic transformation of chronic myeloid malignancies. Am. J. Hematol..

[137] Guglielmelli P., Biamonte F., Score J., Hidalgo-Curtis C., Cervantes F., Maffioli M., Fanelli T., Ernst T., Winkelman N., Jones A.V. (2011). EZH2 mutational status predicts poor survival in myelofibrosis. Blood.

[138] Triviai I., Zeschke S., Rentel J., Spanakis M., Scherer T., Gabdoulline R., Panagiota V., Thol F., Heuser M., Stocking C. (2018). ASXL1/EZH2 mutations promote clonal expansion of neoplastic HSC and impair erythropoiesis in PMF. Leukemia.

[139] Tefferi A., Guglielmelli P., Lasho T.L., Rotunno G., Finke C., Mannarelli C., Belachew A.A., Pancrazzi A., Wassie E.A., Ketterling R. (2014). CALR and ASXL1 mutations-based molecular prognostication in primary myelofibrosis: An international study of 570 patients. Leukemia.

[140] McNamara C.J., Panzarella T., Kennedy J.A., Arruda A., Claudio J.O., Daher-Reyes G., Ho J., Siddiq N., Devlin R., Tsui H. (2018). The mutational landscape of accelerated- and blast-phase myeloproliferative neoplasms impacts patient outcomes. Blood Adv..

[141] Zhang S.-J., Rampal R., Manshouri T., Patel J., Mensah N., Kayserian A., Hricik T., Heguy A., Hedvat C., Gönen M. (2012). Genetic analysis of patients with leukemic transformation of myeloproliferative neoplasms shows recurrent SRSF2 mutations that are associated with adverse outcome. Blood.

[142] Yoshida K., Sanada M., Shiraishi Y., Nowak D., Nagata Y., Yamamoto R., Sato Y., Sato-Otsubo A., Kon A., Nagasaki M. (2011). Frequent pathway mutations of splicing machinery in myelodysplasia. Nature.

[143] Boiocchi L., Hasserjian R.P., Pozdnyakova O., Wong W.J., Lennerz J.K., Le L.P., Dias-Santagata D., Iafrate A.J., Hobbs G.S., Nardi V. (2019). Clinicopathological and molecular features of SF3B1-mutated myeloproliferative neoplasms. Hum. Pathol..

[144] Sheng M.Y., Zhou Y., Xu M.J., Yang F.C. (2014). Role of ASXL1 mutation in myeloid malignancies. Zhongguo Shi Yan Xue Ye Xue Za Zhi/Zhongguo Bing Li Sheng Li Xue Hui J. Exp. Hematol./Chin. Assoc. Pathophysiol..

[145] Zhou Z., Gong Q., Wang Y., Li M., Wang L., Ding H., Li P. (2020). The biological function and clinical significance of SF3B1 mutations in cancer. Biomark. Res..

[146] Aujla A., Linder K., Iragavarapu C., Karass M., Liu D. (2018). SRSF2 mutations in myelodysplasia/myeloproliferative neoplasms. Biomark. Res..

[147] Lasho T.L., Jimma T., Finke C.M., Patnaik M., Hanson C.A., Ketterling R., Pardanani A., Tefferi A. (2012). SRSF2 mutations in primary myelofibrosis: Significant clustering with IDH mutations and independent association with inferior overall and leukemia-free survival. Blood.

[148] Palangat M., Anastasakis D., Fei D.L., Lindblad K.E., Bradley R., Hourigan C.S., Hafner M., Larson D. (2019). The splicing factor U2AF1 contributes to cancer progression through a noncanonical role in translation regulation. Genes Dev..

[149] Visconte V., Nakashima M.O., Rogers H.J. (2019). Mutations in Splicing Factor Genes in Myeloid Malignancies: Significance and Impact on Clinical Features. Cancers.

[150] Tefferi A., Finke C.M., Lasho T.L., Wassie E.A., Knudson R.A., Ketterling R., Hanson C.A., Pardanani A. (2014). U2AF1 mutations in primary myelofibrosis are strongly associated with anemia and thrombocytopenia despite clustering with JAK2V617F and normal karyotype. Leukemia.

[151] Lasho T.L., Finke C.M., Hanson C.A., Jimma T., Knudson R.A., Ketterling R., Pardanani A., Tefferi A. (2012). SF3B1 mutations in primary myelofibrosis: Clinical, histopathology and genetic correlates among 155 patients. Leukemia.

[152] Guglielmelli P., Lasho T.L., Rotunno G., Mudireddy M., Mannarelli C., Nicolosi M., Pacilli A., Pardanani A., Rumi E., Rosti V. (2018). MIPSS70: Mutation-Enhanced International Prognostic Score System for Transplantation-Age Patients with Primary Myelofibrosis. J. Clin. Oncol..

[153] McMullin M.F., Cario H. (2016). LNK mutations and myeloproliferative disorders. Am. J. Hematol..

[154] Maslah N., Cassinat B., Verger E., Kiladjian J.-J., Velazquez L. (2017). The role of LNK/SH2B3 genetic alterations in myeloproliferative neoplasms and other hematological disorders. Leukemia.

[155] Pardanani A., Lasho T.L., Finke C.M., Oh S.T., Gotlib J., Tefferi A. (2010). LNK mutation studies in blast-phase myeloproliferative neoplasms, and in chronic-phase disease with TET2, IDH, JAK2 or MPL mutations. Leukemia.

[156] Rumi E., Harutyunyan A.S., Pietra D., Feenstra J.D.M., Cavalloni C., Roncoroni E., Casetti I., Bellini M., Milanesi C., Renna M.C. (2016). LNK mutations in familial myeloproliferative neoplasms. Blood.

[157] Harutyunyan A., Klampfl T., Cazzola M., Kralovics R. (2011). p53 Lesions in Leukemic Transformation. N. Engl. J. Med..

[158] Courtier F., Carbuccia N., Garnier S., Guille A., Adélaïde J., Cervera N., Gelsi-Boyer V., Mozziconacci M.-J., Rey J., Vey N. (2016). Genomic analysis of myeloproliferative neoplasms in chronic and acute phases. Haematologica.

[159] Grinfeld J., Nangalia J., Baxter E.J., Wedge D., Angelopoulos N., Cantrill R., Godfrey A.L., Papaemmanuil E., Gundem G., MacLean C. (2018). Classification and Personalized Prognosis in Myeloproliferative Neoplasms. N. Engl. J. Med..

[160] Elliott M.A., Pardanani A., Hanson C.A., Lasho T.L., Finke C.M., Belachew A.A., Tefferi A. (2015). ASXL1mutations are frequent and prognostically detrimental inCSF3R-mutated chronic neutrophilic leukemia. Am. J. Hematol..

[161] Nooruddin Z., Miltgen N., Wei Q., Schowinsky J., Pan Z., Tobin J., Purev E., Gutman J.A., Robinson W., Pollyea D.A. (2017). Changes in allele frequencies of CSF3R and SETBP1 mutations and evidence of clonal evolution in a chronic neutrophilic leukemia patient treated with ruxolitinib. Haematologica.

[162] Iurlo A., Gianelli U., Beghini A., Spinelli O., Orofino N., Lazzaroni F., Cambiaghi S., Intermesoli T., Rambaldi A., Cortelezzi A. (2014). Identification of kitM541L somatic mutation in chronic eosinophilic leukemia, not otherwise specified and its implication in low-dose imatinib response. Oncotarget.

[163] Schwaab J., Umbach R., Metzgeroth G., Naumann N., Jawhar M., Sotlar K., Horny H.P., Gaiser T., Hofmann W.K., Schnittger S. (2015). KIT D816V and JAK2 V617F mutations are seen recurrently in hypereosinophilia of unknown significance. Am. J. Hematol..

[164] Wang S.A., Tam W., Tsai A., Arber D.A., Hasserjian R.P., Geyer J.T., George T., Czuchlewski D.R., Foucar K., Rogers H.J. (2016). Targeted next-generation sequencing identifies a subset of idiopathic hypereosinophilic syndrome with features similar to chronic eosinophilic leukemia, not otherwise specified. Mod. Pathol..

